# Chimeric Vaccines Based on Novel Insect-Specific Flaviviruses

**DOI:** 10.3390/vaccines9111230

**Published:** 2021-10-22

**Authors:** Jessica J. Harrison, Jody Hobson-Peters, Helle Bielefeldt-Ohmann, Roy A. Hall

**Affiliations:** 1 Australian Infectious Diseases Research Centre, School of Chemistry and Molecular Biosciences, University of Queensland, St. Lucia, Brisbane, QLD 4072, Australia; j.peters2@uq.edu.au (J.H.-P.); h.bielefeldtohmann1@uq.edu.au (H.B.-O.); roy.hall@uq.edu.au (R.A.H.); 2 School of Veterinary Science, University of Queensland, Gatton, QLD 4343, Australia

**Keywords:** flavivirus, vaccine platform, chimeric virus, dengue, yellow fever, Japanese encephalitis, Zika, West Nile virus disease, insect-specific flavivirus, Binjari virus

## Abstract

Vector-borne flaviviruses are responsible for nearly half a billion human infections worldwide each year, resulting in millions of cases of debilitating and severe diseases and approximately 115,000 deaths. While approved vaccines are available for some of these viruses, the ongoing efficacy, safety and supply of these vaccines are still a significant problem. New technologies that address these issues and ideally allow for the safe and economical manufacture of vaccines in resource-poor countries where flavivirus vaccines are in most demand are urgently required. Preferably a new vaccine platform would be broadly applicable to all flavivirus diseases and provide new candidate vaccines for those diseases not yet covered, as well as the flexibility to rapidly pivot to respond to newly emerged flavivirus diseases. Here, we review studies conducted on novel chimeric vaccines derived from insect-specific flaviviruses that provide a potentially safe and simple system to produce highly effective vaccines against a broad spectrum of flavivirus diseases.

## 1. Introduction

**Figure 1:**
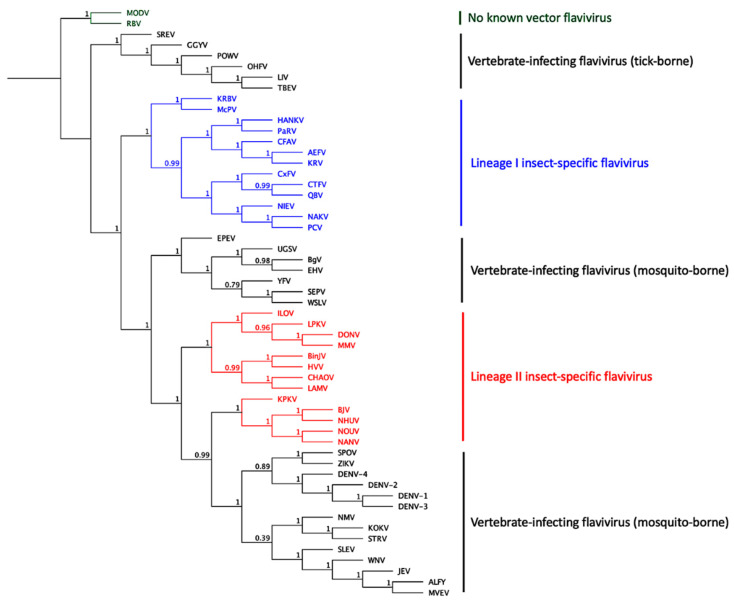
Dendrogram of flavivirus phylogenetic relationships using a maximum-likelihood model and complete amino acid sequences. Sequences were derived using the following GenBank accession numbers: *Aedes* flavivirus (AEFV AB488408), Alfuy virus (ALFV AY898809), Bamaga virus (BgV KU308380), Binjari virus (BinJV MG587038), Barkedji virus (BJV KC496020), cell fusing agent virus (CFAV KJ741267), Chaoyang virus (CHAOV JQ308185), *Culex theileri* flavivirus (CTFV HE574574), *Culex* flavivirus (CxFV AB262759), dengue virus serotype 1 (DENV-1 U88536), dengue virus serotype 2 (DENV-2 U87411), dengue virus serotype 3 (DENV-3 AY099336), dengue virus serotype 4 (DENV-4 AF326825), Donggang virus (DONV NC_016997), Edge Hill virus (EHV DQ859060), Ecuador Paraiso Escondido virus (EPEV NC_027999), Gadgets Gully virus (GGYV DQ235145), Hanko virus (HANKV NC_030401), Hidden Valley virus (HVV MN954647), Ilomantsi virus (ILOV KC734549), Japanese encephalitis virus (JEV NC_001437), Kokobera virus (KOKV AY632541), Kampung Karu virus (KPKV KY320648), Karumba virus (KRBV NC_035118), Kamiti River virus (KRV AY149905), Lammi virus (LAMV KC692068), Louping ill virus (LIV Y07863), Long Pine Key virus (LPKV KY290256), Mac Peak virus (McPV NC_035187), Modoc virus (MODV AJ242984), Murray Valley encephalitis virus (MVEV AF161266), Nakiwogo virus (NAKV NC_030400), Nanay virus (NANV MF139575), Nhumirim virus (NHUV KJ210048), Nienokoue virus (NIEV JQ957875), New Mapoon virus (NMV KC788512), Nounane virus (NOUV EU159426), Omsk hemorrhagic fever virus (OHFV AY193805), Parramatta River virus (PaRV KT192549), Palm Creek virus (PCV KC505248), Powassan virus (POWV L06436), Quang Binh virus (QBV FJ644291), Rio Bravo virus (RBV NC_003675), Sepik virus (SEPV DQ837642), St. Louis encephalitis virus (SLEV DQ525916), Spondweni virus (SPOV DQ859064), Saumarez Reef virus (SREV DQ235150), Stratford virus (STRV KM225263), tick-borne encephalitis virus (TBEV U27495), Uganda S virus (UGSV DQ859065), West Nile virus (WNV KY229074), Wesselsbron virus (WSLV JN226796), yellow fever virus (YFV X03700) and Zika virus (ZIKV AY632535).

The genus *Flavivirus* (family: *Flaviviridae*) comprises over 70 distinct members and is separated into four distinct groups: mosquito-borne (circulating between mosquitoes and vertebrates); tick-borne (circulating between ticks and vertebrates); no known vector (infect vertebrates, but no known arthropod vector has been identified) and insect-specific flaviviruses (which are only found in mosquitoes and cannot infect vertebrates) (Figure 1). The *Flavivirus* genus contains some of the world’s most medically important vector-borne viruses, including yellow fever virus (YFV), dengue virus (DENV), Japanese encephalitis virus (JEV), West Nile virus (WNV) and tick-borne encephalitis virus (TBEV). Despite their geographical limitations and the relatively small reported case numbers, several other species also cause severe or lethal diseases in humans. As such, they still pose a threat as emerging or re-emerging pathogens. Examples include St Louis encephalitis virus (SLEV), Murray Valley encephalitis virus (MVEV), Rocio virus (ROCV), Kyasanur Forest disease virus (KFDV), Omsk hemorrhagic fever virus (OHFV) and Powassan virus (POWV).

Flaviviruses have distinct geographical distributions based on vector species association and vertebrate host range. YFV is transmitted by *Aedes aegypti* mosquitoes, is endemic in tropical and subtropical regions in Africa and South America and causes an estimated 130,000 cases with 78,000 deaths annually [1]. DENV is also transmitted by *Aedes aegypti* and is endemic in the same regions as YFV, as well as in South-East Asia. It causes an estimated 100–200 million infections annually, with approximately 500,000 cases of severe disease (dengue hemorrhagic fever/shock syndrome) and roughly 20,000 deaths [2]. JEV, the most prevalent cause of viral encephalitis in Asia, is transmitted by *Culex* vectors and maintained in avian and porcine vertebrate hosts. It causes an estimated 50,000 cases annually [3] with a 25–30% mortality rate and long-term sequelae in up to 75% of survivors [4]. In contrast, TBEV is endemic in Europe and Central and Eastern Asia and causes an estimated 10,000 infections each year [5]. Morbidity and mortality associated with flaviviral infections may increase as mosquito-borne viruses evolve and expand geographically. Indeed, outbreaks of flaviviral diseases are now occurring in temperate areas [6,7].

WNV is one of the best examples of how an emergent flavivirus can quickly establish itself in previously unaffected areas. WNV was initially endemic in Africa, South-East Europe, Asia and Australia, and occasionally caused sporadic cases of West Nile fever and encephalitis. However, large human outbreaks caused by virulent strains of the virus started to appear in Romania, Russia and the United States of America (USA) in the late 1990s [8,9] and WNV is now the leading cause of mosquito-borne disease in continental USA. Despite this, no WNV vaccine is licensed for human use. Although an effective WNV vaccine remains elusive for humans, successful prevention of WNV has already been achieved for veterinary purposes. Disease in horses decreased after licensing a formalin-inactivated virus vaccine [10] and a canarypox chimeric vaccine [11] in the early 2000s. Several formalin-inactivated veterinary vaccines are currently being used to immunize horses [12,13].

## 2. Flavivirus Vaccine Development

Despite preclinical and clinical trials on a range of experimental flavivirus vaccines, to date, vaccines for humans have only been approved for a few flaviviral pathogens. Traditional flavivirus vaccines include live-attenuated or inactivated whole virus preparations. A significant breakthrough in the history of viral vaccines was the development of the live-attenuated YFV 17D vaccine [14], for which Max Theiler received the Nobel Prize in Physiology/Medicine in 1951. Although efficacious, severe adverse effects caused by vaccination have been reported over the years, with viscerotropic and vaccine-associated neurotropic disease incidents reported in mainly immunocompromised and elderly individuals [15]. Nevertheless, it is still considered a safe and effective vaccine and has continued to be used to vaccinate travellers and people living in endemic areas [16]. Despite the early success of the 17D vaccine, approved live-attenuated or inactivated vaccines derived from native viruses are only widely available for a few flaviviral diseases (Table 1).

## 3. Genetically Engineered Live-Attenuated Vaccines

Improvements in technology and the success and proven efficacy of the YFV 17D vaccine led to the development of several attenuated chimeric vaccine candidates that were based on infectious clone technology [24], where the prM and E genes of YFV were replaced with those of JEV, DENV or WNV (Table 2). The resulting chimeric viruses retained the safety profile of the attenuated 17D parental virus while inducing an immune response to a heterologous flavivirus by authentically displaying the associated structural proteins [25,26,27,28,29]. Indeed, chimeric vaccines for JEV (IMOJEV/THAIJEV) and DENV (Dengvaxia/CYD-TDV) have now been approved for use in humans, while several promising chimeric candidates, including those that express heterologous prME from DEN-2 or DENV-4 backbones, are currently in human clinical trials (Table 2).

## 4. Challenges for Flavivirus Vaccine Development

During the major ZIKV outbreak in South America in 2015, despite relatively mild disease manifestation in children and adults, a new form of disease known as foetal Zika syndrome (FZS) was identified in infants born to women infected during their first trimester of pregnancy. ZIKV was shown to transplacentally infect the foetus, resulting in foetal brain infection and defective neural development [37,38,39,40]. This phenomenon prompted a rapid expansion of research into developing a ZIKV vaccine. While vaccination may be unnecessary to protect individuals from the disease in ZIKV endemic regions due to the rarity of severe disease, it is urgently required for women who are currently or seeking to be pregnant to prevent FZS [41]. Developing a ZIKV vaccine is challenging as live-attenuated vaccines are generally deemed unsafe for administration to pregnant women while inactivated vaccines are not as effective in inducing protective immunity [42,43,44]. Indeed, pregnant women are advised against being vaccinated with the live-attenuated YFV and JEV vaccines since, in most instances, the risk to the pregnancy outweighs the risk of virus exposure [45]. The lack of an effective immune system in the foetus, in combination with the dampened immune response of the pregnant woman, severely increases the probability of endangering the pregnancy if transplacental transmission of any live vaccine components were to occur [46]. It should be noted that during outbreaks and high infection probabilities the risk of infection may outweigh the risk of vaccination. While some chimeric ZIKV vaccine candidates are showing promising results in murine and simian models [47,48], it is unknown if sterilizing immunity is required for fully protecting the mother and foetus [41,49,50]. Higher neutralizing antibody titres against ZIKV are necessary to protect pregnant mice against in-utero transmission than to protect against clinical signs in non-pregnant mice [41,51], likely due to the immune dampened environment in the uterine milieu [52]. Sterilising immunity has been difficult to induce and has not yet been successfully achieved for any flavivirus vaccine [50].

### Induction of Non-Neutralizing Cross-Reactive Antibodies and Antibody-Dependent Enhancement

Flaviviruses possess conserved immunodominant epitopes in the envelope protein that induce variable cross-reactive antibody responses upon infection. While this can be problematic for diagnostic specificity, the most significant challenge it presents is the potential induction of antibody-dependent enhancement (ADE) of infection from viruses such as DENV, leading to severe forms of the disease. This phenomenon is mediated by cross-reacting antibodies from prior infection with one DENV serotype at sub-neutralizing levels. Subsequent infections with a different DENV serotype are enhanced via the increased uptake of virus-antibody complexes into macrophages and monocytes expressing Fc receptors [53,54].

Indeed, severe clinical outcomes following serial DENV infections have made developing a vaccine an elusive global health imperative as effective tetravalent live-attenuated vaccines have proved challenging to develop. While studies have shown that a 30-nucleotide deletion in the 3′-UTR of DENV resulted in no detectable viremia and high levels of neutralizing antibodies after inoculating rhesus macaques (*Macaca mulatta*) [55], trials assessing the tetravalent equivalent have shown that some serotypes in the vaccine cocktail have outcompeted the others, thus not inducing an equal neutralizing antibody response against all serotypes. Despite high post-vaccination neutralizing antibody titres, DENV-3 viraemia was observed in all vaccine recipients, and moderate clinical signs of disease were reported, including fever, maculopapular rash and transient neutropenia [56]. Tetravalent live-attenuated vaccines have since been tested in healthy children and adults, but similar observations and DENV-3 related disease, including fever, were reported [57,58]. Dengvaxia, the only licensed vaccine against DENV, has recently become the subject of controversy. Shortly after licensing, it was found that while Dengvaxia reduced the overall risk of severe dengue and hospitalization, the protection was more apparent in those who had prior history of DENV infection [59]. Thus, those who had never been infected with DENV before may be of increased risk of a more severe form of DENV, than had they not been vaccinated at all [59].

A new tetravalent vaccine developed by Takeda, in which the prM and E genes of each DENV serotype are expressed on a DENV-2 backbone has shown promising results in Phase III clinical trials. The vaccine, TAK-003, was shown to be well-tolerated and efficacious against symptomatic dengue in children regardless of serological status before immunisation. However, the vaccine efficacy varied by serotype, ranging between 48.9 % (DENV-3) and 95.1% (DENV-2), thus longer-term assessments are warranted [35].

In some cases of pre-existing immunity, serum from individuals with high neutralising antibody levels to DENV enhanced ZIKV infection in vitro. However, this hypothesis was unsupported when the phenomenon was further investigated in vivo using a rhesus macaque model [60].

## 5. Insect-Specific Flaviviruses

More recently, a new subgroup of flaviviruses restricted to replicating only in mosquito hosts has been identified. These insect-specific flaviviruses (ISFs) have been detected globally, infecting multiple mosquito genera (Table 3). While the isolation and characterization of ISFs provide essential insights into the evolutionary history of the *Flavivirus* genus, more recent data suggest that some of these viruses can regulate the transmission of vertebrate-infecting flaviviruses (VIFs), such as WNV and ZIKV in co-infected mosquitoes [61,62,63,64,65,66], as well as act as a genetic backbone to produce hybrid viruses that can be used as safe vaccines and diagnostic reagents for flaviviral pathogens [67,68,69,70,71].

The first ISF was discovered in 1975 when Stollar and Thomas sought to investigate the causative agent of cytopathic effects (CPE) in an *Aedes aegypti*-derived cell line [72]. Further characterization of the infectious agent revealed a new virus that shared characteristics with other flaviviruses but did not display antigenic cross-reactivity [73], indicating the presence of a divergent species. Called cell fusing agent virus (CFAV), the new virus was referred to as “insect-specific” as in vitro replication in vertebrate cells was not detectable [72]. The complete genome was not elucidated until 1992 when initial suspicions were confirmed that the new virus belonged to the *Flavivirus* genus [73]. While the first field isolation of CFAV did not occur till 2006, where it was identified at high prevalence in *Aedes aegypti* mosquitoes captured in Puerto Rico, the closely related Kamiti River virus (KRV) was detected and isolated from *Aedes macintoshi* mosquitoes captured in Kenya in 1999 [74]. Many other ISF species have been genetically detected or isolated from different mosquito species in various regions of the world since (Table 3) [75,76] and form two distinct lineages with the *Flavivirus* genus: Lineage I (classical) or Lineage II (dual-host affiliated) ISFs (Figure 1) [75,76].

**Figure 2:**
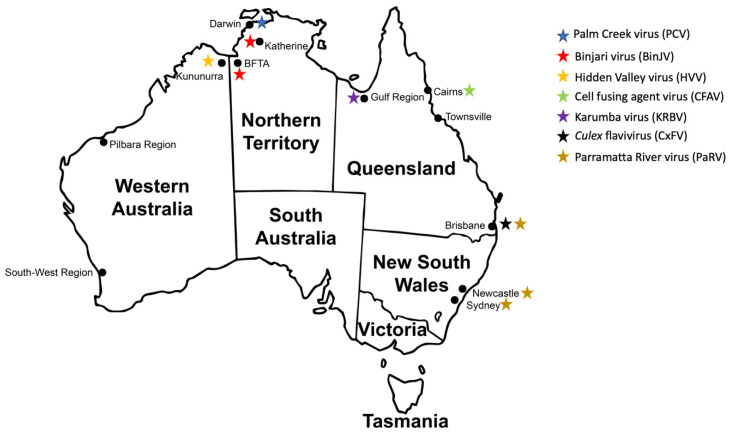
Insect-specific flaviviruses detected and isolated in Australian mosquitoes.

Several ISF species of both lineages have been detected and isolated in Australia. Concerning Lineage I ISFs, Parramatta River virus (PaRV) was isolated from *Aedes vigilax* mosquitoes captured in Sydney, Newcastle and Brisbane [77]. In contrast, Palm Creek virus (PCV) was detected in *Coquillettidia xanthogaster* mosquitoes captured in Darwin in 2010 (Figure 2) [64]. More recently, a new clade of *Anopheles*-associated ISFs, including the Karumba virus (KRBV), has been detected [78], and Australian isolates of CFAV and *Culex flavivirus* (CxFV) have been detected in *Aedes aegypti* and *Culex quinquefasciatus* mosquitoes, respectively (Figure 2) [79,80]. In contrast, only two Lineage II ISFs have been isolated from Australian mosquitoes. Binjari virus (BinJV) was first detected in *Aedes normanensis* mosquitoes captured in Katherine, Northern Territory, in 2010 and subsequently isolated from the same mosquito species caught at the Bradshaw Field Training Area (BFTA), approximately 300 km from Katherine, in 2014 [69]. Hidden Valley virus (HVV) was isolated from archival collections of *Aedeomyia catasticta* mosquitoes that had been captured at Kununurra, Western Australia, in 1975 (Figure 2) [81].

## 6. Construction of Chimeric Flaviviruses Based on ISF Genomic Backbones

**Figure 3:**
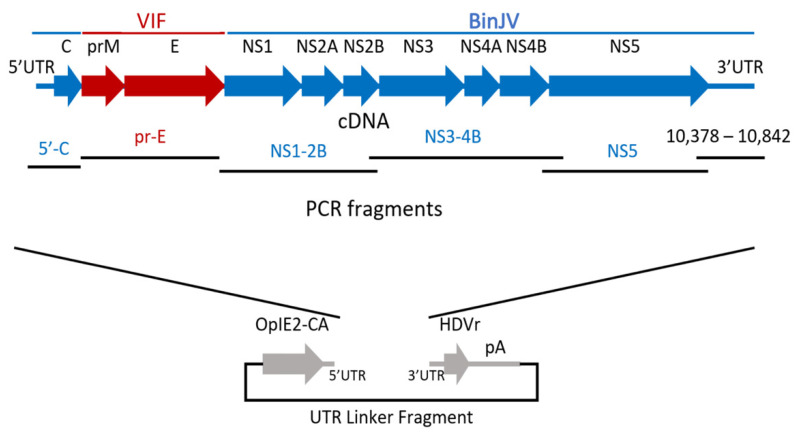
Schematic of the CPER strategy to generate infectious DNA of chimeric BinJ/VIF-prME viruses. BinJ/VIF-prME chimeric viruses are generated by amplifying DNA fragments that share overlapping terminal regions before annealing together in CPER and transfecting mosquito cells with the reaction. Figure adapted from [69].

While we and others have shown that ISFs cannot replicate in a range of vertebrate cells lines [64,69,75,78,81,98,100,105,106,107], we constructed a series of chimeric viruses between ISFs and vertebrate infecting viruses (VIFs) to further analyse the viral factors associated with this host restriction (Figure 3). Using circular polymerase extension reaction (CPER), a novel system that allows for the construction and in vivo transcription of viral cDNA and rescue of infectious RNA viruses without the need for cloning, we generated a series of wild-type and recombinant flaviviruses. These included wild-type Lineage I (PCV) and Lineage II (BinJV) ISFs as well as chimeric viruses between these ISFs and a range of VIFs (e.g., WNV, ZIKV, YFV and DENV; Table 4) [67,68,69,70,71,81,107,108]. This new system provided the tools to investigate mechanisms of host restriction and platforms for rapidly generating recombinant and mutant RNA viruses [109].

## 7. Host Range Restriction of ISFs

Although the molecular mechanisms behind ISF host restriction are not fully elucidated, several barriers to ISF replication in vertebrate cells have been identified. For Lineage I ISFs, independent studies using chimeric viruses expressing the structural genes of Lineage I ISFs Nienokoune virus (NIEV) or PCV and the non-structural genes and untranslated regions (UTRs) of YFV or WNV Kunjin subtype (WNV_KUN_) concluded that a blockage of ISF replication in vertebrate cells occurred pre-entry when infectious particles were not recovered following vertebrate cell infection [98,107]. The generation of a NIEV replicon established that blocks also happened at the level of RNA replication and during virus assembly/release [98]. In addition, the failure of a chimeric virus expressing the structural genes of WNV_KUN_ and the replicative genes and UTRs of PCV to initiate replication in vertebrate cells, including cells lacking an intact interferon response, indicated that additional barriers to ISF replication occur post-entry and are independent of the interferon response [108].

Similar host restriction studies have been conducted with the Australian Lineage II ISF, BinJV. Trace levels of BinJV replication were consistently detected in BSR cells when infected with a multiplicity of infection (MOI) of 50 and incubated at 34 °C, suggesting that in contrast to Lineage I ISFs, the structural genes of Lineage II ISFs were able to facilitate entry to vertebrate cells, albeit inefficiently [81]. Likewise, trace levels of replication of WNV_KUN_/BinJV-prME could be detected in some cultures of mouse embryo fibroblasts (MEFs) deficient in antiviral responses (IFNAR^−/−^ MEFs or RNase L^−/−^ MEFs) incubated at 34 °C post-inoculation [81,110]. In contrast to our findings using the WNV_KUN_/BinJV-prME chimera, data generated by inoculating Vero cells with chimeric viruses containing the prM and E genes of Lineage II ISF Long Pine Key virus on a ZIKV backbone suggested that vertebrate cells support virus translation but not growth [111]. However, this study did not assess the effect on viral replication at a lowered incubation temperature, which can affect flaviviral replication [81,110,112,113]. In light of this, the ability of Lineage II ISF structural genes facilitating entry into vertebrate cells should be assessed further.

Wild-type BinJV and BinJ/VIF-prME chimeras failed to initiate replication in a wide range of vertebrate cell lines at 37 °C, including cells lacking components of the innate immune response. This observation indicates that BinJV replication in vertebrate cells is inhibited at temperatures above 36 °C, as observed for other flaviviruses [112,113,114], and is restricted at multiple stages of cellular infection, including inefficient cell entry and susceptibility to antiviral responses. In contrast to the BinJV studies, in vitro assessment of Lineage II ISFs LAMV and ILOV replication in a range of vertebrate cells incubated at different temperatures failed to detect replication [100]. However, this may be due to a low MOI used in the study. In vivo studies also failed to detect LAMV and ILOV RNA in the brains of intracerebrally-inoculated suckling mice [100]. Similarly, inoculation of high titres of BinJ/ZIKV-prME virus into immunocompromised mice (IFNAR^−/−^ and NRG [NOD.Cg-Rag1^tm1Mom^Il2rg^tm1Wjl^/Szj]) failed to result in detectable levels of viral genome amplification [69].

The lack of efficient ISF replication in vertebrate cell lines, even at lower temperatures, high MOI and immune-deficient cell lines, suggests that additional barriers inhibit steps in the virus replication cycle and are potentially mediated by cellular antiviral responses. However, this remains to be tested in primary cell cultures. Our recent studies incriminate the zinc-finger antiviral protein (ZAP) as a crucial player in this context [110]. It appears that the high frequency of CpG dinucleotides in the ISF genome sequence, that are not observed in the genome of VIFs, enables ZAP to specifically bind the ISF RNA genome and target it for degradation, thus inhibiting replication [115]. Furthermore, when wild-type BinJV and chimeric BinJ/WNV_KUN_-prME were cultured in wild-type human A549 cells and ZAP-knockout A549 cells at 34 °C, replication was only detected in the ZAP-KO cells [110].

## 8. Antigenic and Structural Analysis of Chimeras

To demonstrate that chimeric ISF particles authentically display the structural proteins of VIFs on their surface, BinJ/WNV_KUN_-prME, BinJ/ZIKV-prME, BinJ/DENV2-prME were compared to their wild-type VIF (WT VIF) counterparts (WNV_KUN_, ZIKV or DENV-2) using a range of antibody-binding assays including ELISA and IFA [69]. An extensive panel of mAbs targeting the prM or E proteins of each VIF were titrated and tested for binding to either BinJ/VIF-prME or the corresponding WT VIF in ELISA to generate apparent dissociation (K_d_) curves. In each instance, the K_d_ correlation between chimeric virus and parental virus was high, with lines of best fit developing R_2_ values of 0.998 (BinJ/WNV_KUN_-prME), 0.930 (BinJ/ZIKV-prME) and 0.886 (BinJ/DENV2-prME), indicating high levels of antigenic authenticity in the structural proteins of BinJ/VIF-prME chimeras [69]. Furthermore, the structures of these chimeric ISF particles were very similar to corresponding WT VIFs when gradient purified viral preparations were analysed by high-resolution cryo-electron microscopy. Even at very high resolutions, BinJV/WNV_KUN_-prME particles (3.9Å) and BinJV/ZIKV-prME particles (7.1Å) were indistinguishable from the virions of their WT VIF counterparts [69,116].

## 9. Use of Insect-Specific Flaviviruses as Vaccine Antigens for Pathogenic Flaviviruses

The demonstration that chimeric viruses, based on the insect-specific alphavirus Eilat virus, could be used as safe and effective experimental vaccines against alphavirus pathogens such as chikungunya virus (CHIKV), Eastern equine encephalitis virus and Venezuelan equine encephalitis virus [117,118], also provided precedence to assess ISF-based chimeras for this purpose. Based on the high safety profile of BinJ/VIF-prME chimeric viruses (i.e., a lack of replication in vertebrate cells), and the structural and antigenic authenticity of their virions, we further assessed their ability to induce protective immune responses to wild-type flaviviral pathogens after immunisation in the animal models of these diseases. This study involved the construction and assessment of BinJV chimeras containing the prM-E genes of ZIKV, YFV, DENV and WNV.

### 9.1. Vaccine Efficacy against ZIKV

Using an immunodeficient mouse model of ZIKV disease (IFNAR^−/−^), vaccination with one or two small unadjuvanted doses of purified BinJ/ZIKV-prME chimera (2 or 20 µg) induced robust neutralizing antibody responses against wild-type ZIKV (ZIKV_PVRABC59_ strain) and protected against infection (viraemia) and pathology (weight loss or tissue pathology) after challenge with wild-type ZIKV (Figure 6 in [69]). This protection was also extended to the foetus in a pregnant mouse model of ZIKV disease, after a similar vaccination with BinJ/ZIKV-prME, with no evidence of induction of antibodies that mediate enhancement of DENV-2 virus in Fc expressing cells [68].

### 9.2. Vaccine Efficacy against WNV

Efficacious protection was also demonstrated against a lethal WNV challenge when mice were vaccinated with BinJ/WNV_KUN_-prME. When 6–8-week-old CD1 mice were immunised with low doses (1 or 5 µg) of unadjuvanted, purified BinJ/WNV_KUN_-prME, a single dose was sufficient to induce a robust neutralizing antibody response against both WNV_KUN_ (NSW_2011_ strain) and the more virulent WNV_NY99_ strain [70]. Furthermore, this response provided complete protection against viremia and mortality when the mice were challenged with a lethal dose of WNV_NY99_, with no clinical pathology observed in vaccinated mice [70].

### 9.3. Vaccine Efficacy against DENV

AG129 mice immunized with a BinJ/DENV2-prME chimera were similarly completely protected against lethal challenge with a mouse adapted strain of DENV-2 (DENV2, D220) [67]. In these studies, the protective efficacy of the chimera was also assessed by delivery to the skin via a high-density microarray patch (HD-MAP), a method which has previously been shown to elicit induction of enhanced immune responses with significant dose sparing [119,120,121,122]. When delivered by HD-MAP, mice vaccinated with a single 1 μg dose of BinJ/DENV2-prME (without adjuvant) had significantly reduced levels of virus and NS1 loads in the blood after DENV-2 challenge when compared to unvaccinated mice and were fully protected [67]. Studies assessing BinJV chimeras expressing prME of the remaining three DENV serotypes are currently underway.

### 9.4. Vaccine Efficacy against YFV

Vaccination of female IFNAR^−/−^ mice with two 5 µg doses of BinJ/YFV_17D_-prME induced neutralizing antibodies and protected mice against infection, weight loss and liver pathology after YFV 17D challenge [71]. In contrast to the BinJV-derived chimeric vaccines described above, BinJ/YFV-prME required the addition of an adjuvant (AS01) to induce protection. AS01 is a liposome-based adjuvant that contains two immunostimulants: 3-*O*-desacyl-4′-monophosphoryl lipid A, a non-toxic derivative of the lipopolysaccharide from *Salmonella minnesota*; and QS-21, a saponin fraction extracted from *Quillaja saponaria* Molina [123]. The reason for the lower efficacy of BinJ/YFV_17D_-prME compared to the other BinJ/VIF-prME vaccines, which did not require an adjuvant, is not clear.

## 10. BinJ-VIF Chimeric Vaccines Are Highly Immunogenic

As discussed above, except for the BinJ/YFV_17D_ chimeric vaccine, the BinJ/VIF-prME vaccines described above did not require multiple doses or even an adjuvant to induce protective immunity in the mouse model. A significant contributor to this potent immunogenicity is likely the structural and antigenic authenticity of the chimeric particles, compared to their WT VIF counterparts, as demonstrated by the high-resolution cryo-EM analysis and mAb Kd binding studies discussed earlier. By comparison, many chemically inactivated or recombinant preparations of VLP vaccines have significantly reduced immunogenicity due to the loss of structural and antigenic integrity of the particles [124,125]. Thus, they usually require multiple doses and the addition of adjuvants for robust induction of protection.

Nevertheless, this potent immunogenicity is unusual for vaccines that do not replicate in vertebrate cells and indicates the chimeric virus particles may have self-adjuvanting properties. One possibility is that the chimeric particles enter susceptible cells after vaccination by binding to cellular receptors via the VIF structural proteins on the chimeric particle surface. Subsequently, early stages of viral replication may be initiated, and RIG-I recognition of viral dsRNA intermediates then mediate the production of inflammatory cytokines (e.g., TNF alpha; IL6, etc.) that enhance the humoral response [126,127]. Although productive replication of the BinJ/VIF-prME chimeras is ultimately inhibited by cellular antiviral responses (e.g., ZAP and ISGs) and other factors (37 °C temperature) before the infectious progeny virus is produced, the innate responses to early stages of entry and replication may provide the adjuvanting effect.

To test this theory, we completely inactivated BinJ/WNV_KUN_-prME by UV treatment and compared its protective efficacy with untreated particles by immunizing and challenging with a lethal dose of WNV. Although neutralizing titres were slightly lower in mice receiving the inactivated particles compared to mice receiving the live virus, this treatment did not significantly affect vaccine efficacy with all mice protected from disease after a single unadjuvanted dose [70]. This result suggests that an initial stage of replication of the chimeric vaccine in cells of vaccinated animals is not solely responsible for the potent immunity observed. However, we cannot ignore the possibility that trace contaminants from the culture system (media or mosquito cell components) may have been co-purified with the vaccine particles and provide the adjuvanting effect. Indeed, the N-linked glycans on the surface glycoproteins of flavivirus particles produced in mammalian cells are slightly different from those produced in insect cells. The former contain predominantly complex glycans or mixtures of high-annose and complex glycans, while the latter contain predominantly high mannose-glycans [128,129]. Since glycosylation type may influence the immune response, a possible role for insect-derived high-mannose glycosylation of chimeric particles in their enhanced immunogenicity should be explored.

## 11. Safety and Regulatory Issues of ISF-Based Vaccines

During licensing of live-attenuated vaccines, consideration should be given to the risks involved in the uncontrolled release of the vaccine virus into the environment. This risk is particularly pertinent to genetically modified (GM) vaccine viruses. Because mosquitoes can transmit wild-type flaviviruses, the possibility of vaccine virus transmission by these arthropod-borne vectors should be investigated. For example, the recombinant, live-attenuated Chimeri-Vax vaccines were shown to be highly attenuated in mosquitoes. Although low infection levels were observed in the midgut following a blood meal, these vaccine viruses were not transmitted by mosquitoes as no dissemination through the mosquito occurred [130,131,132,133]. We are similarly assessing the risk that BinJV chimeric viruses pose to the environment.

Some jurisdictions may have strict regulatory requirements for the intentional release of GMOs and may not accept vaccination of humans and animals with a “live”, genetically modified vaccine. Thus, inactivation methods to eliminate the replication of the chimeric virus in mosquito cells may be required. As discussed above, the inactivation of BinJ/WNV_KUN_-prME with UV-C did not reduce its efficacy as a vaccine in the mouse model. No difference was observed in the binding of a panel of mAbs to the UV-inactivated particles compared to the non-treated vaccine preparation [70], suggesting the treatment did not alter epitope presentation. These data indicate that UV-C is a suitable method to inactivate replication-competent RNA in BinJ/VIF-prME, without significantly compromising the vaccine’s protective efficacy and may represent a viable alternative to preparing safe flavivirus vaccines. We are also investigating other inactivation protocols that comply with international biosafety and therapeutic standards (e.g., gamma irradiation) as a simple alternative method to inactivate these chimeric vaccines to remove the GMO classification.

## 12. Future Challenges for ISF-Based Vaccines

The BinJV chimeric viruses described here are potentially an ideal vaccine platform. These viral particles are antigenically indistinguishable from wild-type vertebrate-infecting viruses, replicate to high titres in mosquito cells and fail to replicate in vertebrate cells under standard conditions. However, live-attenuated and killed vaccines must be grown in GMP-approved cell lines or embryonated chicken eggs that meet strict guidelines. To date, the BinJ/VIF-prME chimeras have only been shown to grow efficiently in the C6/36 mosquito cell line, which is not GMP-approved. Proteins in mosquito saliva are known to aggravate the reaction of some individuals to mosquito bites and enhance the immune response [134]. Indeed, many mosquito proteins have been identified as potent allergens in humans that can induce IgE-mediated reactions [135]. While this leads mostly to local cutaneous responses [131,132], severe anaphylaxis may also occur [136,137]. It is therefore imperative to ensure that significant levels of mosquito proteins are not co-purified with the vaccine.

To address these issues with the chimeric alphavirus vaccine candidate (EILV/CHIKV), which is also grown in *Aedes albopictus* cells (C7/10 line), mass spec analysis of purified preparations detected only low levels of three mosquito proteins, which were not linked to known mosquito allergens and are likely to be integral components of alphavirus particles [138]. Additional toxicity studies with this vaccine in mice revealed that animals exposed to sequential *Aedes albopictus* bites every two weeks before being injected with purified EILV/CHIKV showed only minor inflammation at the inoculation sites [138]. These data are promising and indicate that purified preparations of chimeric viruses grown in mosquito cells may not induce hypersensitivity reactions in animals previously exposed to mosquito bites. Similar studies are required for the BinJ/VIF-prME chimeras described in this review.

## 13. Application of ISF-Based Chimeric Viruses for Diagnostics

The antigenic authenticity of the BinJ/VIF-prME chimeric viruses compared to wild-type VIF particles described above also demonstrates this platform is ideal for producing safe, accurate diagnostic antigens against virulent pathogens, even those that generally require a high level of biocontainment (BSL3 or higher). Indeed, the apparent utility of the chimeric viruses has been demonstrated using immune sera in various ELISA formats, microsphere immunoassays and virus neutralization assays showing similar results between chimeric and wild-type derived VIF antigens [67]. Further application of these chimeric antigens to rapid, point-of-care diagnostic devices, such as strip-based lateral flow assays, suitable for use in basic medical facilities, is another promising development in our laboratory.

## 14. Conclusions

The ability to safely grow BinJ/VIF-prME chimeras to high titres in facilities with minimal biocontainment conditions provides a simple platform for preparing antigenically authentic particles as vaccines or diagnostics for globally important flavivirus pathogens such as YFV, ZIKV, DENV and WNV. This platform is highly amenable for its adoption in resource-poor countries that bear the heavy burdens of these diseases and urgently need a continued supply of inexpensive vaccines and diagnostic reagents, but have limited capacity for vaccine manufacturing.

## Figures and Tables

**Table 1:** Live-attenuated and inactivated-whole flavivirus vaccines that are currently approved for use in humans.

Disease	Vaccine	Type of Vaccine	Where Licensed
yellow fever [17]	17D-204 and 17DD	Live attenuated	globally
tick-borne encephalitis [18]	FSME-IMMUNE, Encepur, TBE-MOSCOW, EnceVir, CIBP	Formalin inactivated	European countries
Japanese encephalitis [19,20,21,22]	SA14-14-2	Live-attenuated	China and some Asian countries
	IXIARO/IC51	Formalin inactivated	globally
	JEBIK V	Inactivated	Japan
	ENCEVAC	Inactivated	Japan
	JEEV	Inactivated	India
Kyasanur forest disease [23]	-	Formalin inactivated	India and nearby regions

**Table 2:** Examples of genetically engineered live-attenuated vaccines.

Disease	Parental Viruses	Vaccine	Comments
JEV	YFV 17DD genomic backbone; prM-E of JEV (SA14-14-2)	ChimeriVax-JE (IMOJEV, THAIJEV)	Showed robust protection against virulent JEV challenge in mice and Rhesus macaques [24,30,31,32,33]. Approved for use in humans in Australia, New Zealand, Thailand, Korea, Malaysia, Philippines and Vietnam
WNV	YFV 17DD genomic backbone; prM-E of WNV (NY99)	ChimeriVax-WN	Highly immunogenic and protective in preclinical animal studies [25,27], progressed to Phase II human clinical trial
	YFV 17DD genomic backbone; prM-E of WNV (NY99)	ChimeriVax-WN02 (PreveNile)	Induced high levels of neutralising antibodies after a single dose during clinical trials and was licensed for veterinary use in 2006 but was recalled in 2010
	Dengue 4 (Caribbean 814669) genomic backbone; prM-E of WNV (NY99)	WN-DEN4	Shown to be attenuated in monkeys, induced moderate-to-high neutralising antibody titres and prevented viraemia after WNV challenge [34], progressed to Phase II human clinical trial
DENV	YFV 17DD genomic backbone; prM-E of each DENV serotype (tetravalent)	Dengvaxia, CYD-TDV	Induced long-lasting, cross-neutralising antibody response to all 4 DENV serotypes [30]. Licensed for use over the age of 9 in DENV-endemic areas.
	DENV2 genomic backbone; prM-E of each DENV serotype (tetravalent)	TAK-003 (Takeda)	Shown to be well tolerated and immunogenic against all four serotypes in Phase I and Phase II clinical trials [35]. Ongoing Phase III clinical trials [35,36].

**Table 3:** Geographic distribution and host range of insect-specific flaviviruses ^1^.

Virus	Lineage	Geographic Distribution	Host Range
Cell fusing agent virus—CFAV [82,83,84]	I	USA ^2^, Puerto Rico, Australia ^2^, Indonesia, Mexico, Thailand	*Aedes aegypti*, *Aedes albopictus*, *Culex* species
Kamiti River virus—KRV [74]	I	Kenya	*Aedes macintoshi*
*Aedes* flavivirus—AeFV [82,85,86,87,88,89]	I	Japan, Italy, USA, Thailand ^2^	*Aedes albopictus*, *Aedes flavopictus*
*Culex* flavivirus—CxFV [88,90,91,92,93,94,95,96,97]	I	Japan, Indonesia, China, Guatemala, USA, Mexico, Trinidad, Uganda, Argentina, Australia	*Culex* species
Nienokoune virus—NIEV [98]	I	Cotê d’Ivoire	*Culex* species
Barkedji virus—BJV [99]	II	Senegal, Israel	*Culex perexiguus*
Lammi virus—LAMV [100,101]	II	Finland	*Aedes cinereus*
Marisma mosquito virus—MMV [102,103,104]	II	Spain, Italy	*Aedes caspius*
Ilomantsi virus—ILOV [100]	II	Finland	*Ochlerotatus riparious*, *Anopheles* species
Long Pine Key virus—LPKV [105]	II	USA	*Anopheles crucians*, *Aedes atlanticus*, *Culex nigripalpus*

^1^ This does not represent an exhaustive list of insect-specific flaviviruses. ^2^ Isolated from laboratory colonies.

**Table 4:** Published ISF/VIF-prME chimeric viruses generated by CPER.

ISF Backbone	VIF prME Insert
PCV [107,108]	WNV_KUN_ (MRM61C)
	DENV-2 (NGC)
	ZIKV (Natal)
BinJV [69,81]	WNV_KUN_ (MRM61C, NSW_2011_)
	ZIKV (Natal)
	DENV 1, 2 and 4 (NGC, ET243, ET288)
	YFV (17D)
	JEV (Nakayama)
